# Motivation Modulates Visual Attention: Evidence from Pupillometry

**DOI:** 10.3389/fpsyg.2013.00059

**Published:** 2013-02-12

**Authors:** Agnieszka Wykowska, Christine Anderl, Anna Schubö, Bernhard Hommel

**Affiliations:** ^1^Ludwig Maximilian UniversityMunich, Germany; ^2^Leiden UniversityLeiden, Netherlands; ^3^Goethe UniversityFrankfurt, Germany; ^4^Philipps UniversityMarburg, Germany

**Keywords:** visual attention, action, perception and action, pupillometry, grasping, pointing, movement, action planning

## Abstract

Increasing evidence suggests that action planning does not only affect the preparation and execution of overt actions but also “works back” to tune the perceptual system toward action-relevant information. We investigated whether the amount of this impact of action planning on perceptual selection varies as a function of motivation for action, which was assessed online by means of pupillometry (Experiment 1) and visual analog scales (VAS, Experiment 2). Findings replicate the earlier observation that searching for size-defined targets is more efficient in the context of grasping than in the context of pointing movements (Wykowska et al., 2009). As expected, changes in tonic pupil size (reflecting changes in effort and motivation) across the sessions, as well as changes in motivation-related scores on the VAS were found to correlate with changes in the size of the action-perception congruency effect. We conclude that motivation and effort might play a crucial role in how much participants prepare for an action and activate action codes. The degree of activation of action codes in turn influences the observed action-related biases on perception.

## Introduction

Human attention is traditionally considered a mechanism that allows prioritizing the processing of information that is behaviorally or emotionally relevant (e.g., Hansen and Hansen, 1988; Öhman et al., 2001; Feldmann-Wüstefeld et al., 2011), task-relevant (Folk et al., 1992; Wolfe, 1994; Müller et al., 2009; Wykowska and Schubö, 2010, 2011), signaled to be potentially relevant (Posner, 1980; Müller and Rabbitt, 1989; Friesen and Kingstone, 1998), or simply salient (e.g., Itti and Koch, 2000; Theeuwes, 2010). However, almost none of the available attentional theories consider the further use of the attentional mechanisms beyond perceptual judgment and decision-making. And yet, recent evidence suggests that attentional processes play a major role in action control, that is, in the processes that are following perception and action selection (e.g., Bekkering and Neggers, 2002; Fagioli et al., 2007; Wykowska et al., 2009). For instance, Fagioli et al. (2007) demonstrated that preparing for a manual reaching movement facilitates the detection of location-defined visual oddball stimuli while preparing for a manual grasp facilitates detection of size-defined oddball stimuli. Along similar lines, Wykowska et al. (2009) showed that preparing for a particular action might sensitize the perceptual system to information suited to guide that action. Biasing perception toward action-relevant dimensions would make it easier for motor-control operations to identify the perceptual parameters suited to specify the open parameters of online control, such as hand aperture (Hommel, 2010).

**Figure 1:**
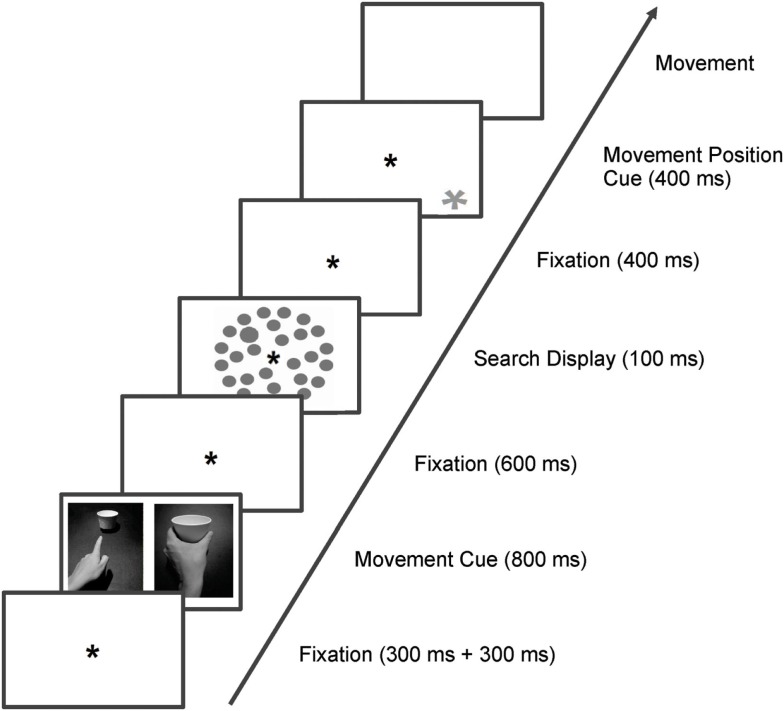
**Trial sequence of Experiment 1 and 2**. Trials started with a fixation mark (in Experiment 1 it was the continuous valid pupil signal of 300+ 300 ms), followed by one of the cues (pointing/grasping; 800 ms), which informed participants which movement they should prepare. After another fixation mark (600 ms), the search display (target/no target) appeared on the screen (100 ms), and was followed by another fixation mark. Four hundred milliseconds after response to the search task, the movement position cue (400 ms) appeared and participants performed the prepared movement on the respective paper cup.

In the paradigm used by Wykowska et al. (2009, see also Wykowska et al., 2011, 2012) participants had to first prepare for a grasping or a pointing movement (as indicated by a cue picture representing a grasping/pointing hand), then detect and report a target in a visual search display (size or luminance pop-out item), and only then carry out the prepared movement on an indicated object (see Figure 1, which depicts an adapted version of the task used in Wykowska et al., 2009). Importantly, the movement task and the visual search task were perceptually and motorically unrelated: the visual search display was presented on a computer screen and the response was to be made on a mouse key with the dominant hand while the movement was to be executed with the other hand on one of the items of a movement execution device (Wykowska et al., 2009, 2011) or on one of three cups positioned below the computer screen (Wykowska et al., 2011, 2012). The design consisted of two action-perception congruent pairs: grasping and size (visual search target defined by size) and pointing and luminance (visual search target defined by luminance), as it was assumed that size is a potentially relevant dimension for a grasping movement while luminance is related to localizing – which is inherently linked to pointing. Results showed action-perception *congruency effects*: detection of a given dimension was facilitated when a congruent movement was being prepared, relative to the incongruent movement. In more detail, detection of size targets was faster when grasping movement was prepared, as compared to the pointing movement; and the reverse pattern was observed for detection of luminance targets. The authors concluded that visual selection is biased by a so-called *intentional weighting* mechanism (Wykowska et al., 2009, 2012; Hommel, 2010; Memelink and Hommel, in press), which prioritizes perceptual processing in order to deliver potentially action-relevant perceptual dimensions for open parameters of online action control, such as hand aperture (Hommel, 2010). Given that in the paradigm of Wykowska and colleagues the movement object was indicated only after the search task, all parameters of the prepared action could not be fully specified before the search task. Therefore, the intentional weighting mechanism prioritized processing of those perceptual dimensions that might have been necessary for efficient online action control.

At the same time, however, in the paradigm of Wykowska and colleagues, preparing the whole action plan was not strictly necessary immediately after the movement cue. Therefore, the less motivated participants might have “kept in mind” what action to do and might have engaged in (more complete) preparation only after completing the search task. This strategy would be expected to reduce or prevent action control processes from taking place before the onset of the visual search display, which should reduce or eliminate congruency effects. The aim of the present study was to characterize the role of action control in visual attention by exploiting individual differences in motivation and effort invested in the task. Re-analyses of data from pilot studies (Anderl, 2009), together with informal observations, have suggested considerable individual differences not so much with respect to the initial motivation for the task but in the maintenance of motivation during the experimental session. A loss of motivation, we reasoned, would be likely to affect the (effortful) preparation of the movement, which in turn could affect action-perception *congruency effects*. Therefore, in the present study, we predicted that individuals with a greater loss of motivation/effort should show a (more) reduced effect of congruency between to-be-prepared action and target dimension.

To provide a reliable but unobtrusive measure of the individual motivational level (so to avoid any impact of the act of measurement on the participants’ motivational state) and its possible change over time, we recorded *tonic* pupil size. The sympathetic nervous system is known to both modulate pupil size (Loewenfeld, 1993) and regulate arousal, so that pupil diameter has often been taken to reflect motivation for, or effort spent on, a task (Ahern and Beatty, 1979; see also Steinhauer and Hakerem, 1992). Indeed, pupil size has been shown to be highly correlated with the level of cognitive effort, with more effort (due to task demands) being reflected in a larger pupil diameter (Hess and Polt, 1964; Beatty and Kahneman, 1966; Loewenfeld, 1993; see Beatty, 1982 for review on pupillometry as a measure of task-related mental effort; Kahneman, 1973 on the idea of effort theory of attention relating task demands to pupil dilation; and Granholm and Steinhauer, 2004 on pupillometry as measure of normal and abnormal cognitive processes). Moreover, changes in pupil diameter have been associated also with shorter-term changes in motivation, as induced by performance-based reward (e.g., Heitz et al., 2008).

Even though most studies have concentrated on *phasic* changes in pupil diameter related to a given task/stimulus, and have indicated that pupil dilation is related to cognitive effort in many domains such as lexical decision (Kuchinke et al., 2007), attention allocation (Karatekin et al., 2004), or load on attentional capacity (Kahneman, 1973), working memory load (Granholm et al., 1996; Van Gerven et al., 2004), or face perception (Goldinger et al., 2009), *tonic* pupil size has also been found to be an indicator of mental effort and arousal (Kahneman, 1973; Gilzenrat et al., 2010; see Laeng et al., 2012 for review), alertness and fatigue (Lowenstein and Loewenfeld, 1962; Merritt et al., 2004), or control state (Gilzenrat et al., 2010).

## Experiment 1

### Materials and methods

#### Participants

Fifteen university students aged from 18 to 32 years participated in this study (age: *M* = 23.3, six males, one left-handed) for partial fulfillment of course credit or a financial reward. All of them reported normal or corrected-to-normal vision. Two participants were excluded from the analysis of pupil data (due to technical problems during recording) but remained in all behavioral analyses. APA ethical standards were followed throughout the study. The experiment was undertaken with the understanding and consent of each participant.

#### Apparatus and stimuli

Stimuli were presented on a standard 17′′ TFT monitor of a remote eye tracker system (Tobii T120, Tobii Technology, Stockholm, Sweden) with a refresh rate of 75 Hz. Stimulus presentation was controlled by E-Prime presentation software (Psychology Software Tools, Pittsburgh, PA, USA).

Participants were seated in central position relative to the midpoint of the screen. Head positions were stabilized with a chin rest at a viewing distance of approximately 50 cm. Room illumination was kept at the level of 100 lux. An asterisk (0.7° of visual angle, presented at the central position of the screen) served as fixation mark. The type of movement required in a trial was indicated by a cue (see Figure 1), which was a black and white photograph (18.4° × 23.7° of visual angle), showing a left hand performing a pointing or a grasping movement of a white paper cup. These cues were also presented centrally on the screen.

The search display (see Figure 1) consisted of 28 gray circles (2.4° of visual angle), which were presented on a white background. They were positioned on three imaginary circular arrays with diameters of 10.4°, 14.1°, and 17.7° of visual angle. Targets were defined as larger circles (3.3° of visual angle) and could appear at the lateralized positions (three left, three right) of the middle circle. Target present trials and target absent trials were randomly intermixed but were presented with equal probability (50%) each. Note that we used only one target dimension (size) to simplify the design for this experiment (similarly to Wykowska et al., 2011; Wykowska et al., 2012, Experiment 1), although the first studies of Wykowska et al. (2009) showed congruency effects for both size and luminance targets when the dimensions were blocked. As size is a relevant perceptual dimension for grasping but not for pointing movements, trials with grasping cues will be referred to as congruent whereas trials with pointing cues will be referred to as incongruent.

For each trial, the movement-relevant cup was indicated by a yellow asterisk (1.4° of visual angle; CIE L*a*b color coordinates: 87/5/82), which could appear at one of three different positions on the screen (10.0° of visual angle below the imaginary midline of the screen in vertical direction and −11.6°, 0°, and 11.6° of visual angle measured from the midline of the screen in horizontal direction). The positions were randomly intermixed and equally likely (33.3% each).

Directly below the three possible positions of the yellow asterisk, three white paper cups were positioned on a board that was installed at 20 cm below the computer screen. Participants were instructed to perform the prepared movement (pointing or grasping) on the indicated paper cup. The cups were identical in height (6.2 cm) but differed in diameter measured at 3.1 cm height (small: 5.3 cm, medium: 6.6 cm, large: 7.6 cm). Positions of the cups (left, middle, right position) were randomized between participants.

Participants were to indicate whether or not they had detected a target by pressing a mouse key with the index and middle finger of their dominant hand. The assignment between mouse keys and target present/absent trials was balanced between participants. The movement task (pointing vs. grasping of a cup) was carried out with the non-dominant hand to allow for simultaneous movement preparation (non-dominant hand) and response to the search task (dominant hand). The experimenter monitored the performed movements with a camera and coded their correctness online with a mouse key.

#### Procedure

Participants attended a 30-min practice session to become familiar with the movement task (180 trials). The experiment proper was conducted no earlier than 2 h and no later than 2 days after the practice session. It started with a five-point calibration and validation procedure of the eye signal. Subsequently, two practice (80 trials each) and two experimental blocks (240 trials each) were performed.

Trial sequence and timing are depicted in Figure 1. Trials started with a fixation asterisk (continuous valid pupil signal of 300 + 300 ms), followed by one of the cues (pointing/grasping; 800 ms), which informed participants which movement they should prepare. After another fixation mark (600 ms), the search display (target/no target) appeared on the screen (100 ms), and participants were supposed to respond to the visual search display as fast as possible. Those speeded responses to the search display were given by pressing the left or right mouse key for target present or absent trials respectively (or vice versa). Reaction times were measured as the time between the onset of the visual search display and key press. Upon the visual search response, another fixation mark was presented for 400 ms. Subsequently, an asterisk signaling which object to grasp/point to (400 ms) appeared, and participants performed the prepared movement on the respective paper cup. The movement task was not speeded but accuracy was stressed. Each trial ended with the registration of the movement type by the experimenter, followed by a 100-ms intertrial interval. Importantly, participants were instructed to prepare for the movement indicated by the cue but not to perform it until one of the yellow asterisks appeared on the screen to indicate the movement’s object. This was done in order to make sure that the movement representation would be active while participants were performing the visual search task.

### Data analysis^1^

#### Behavioral analysis

For RT analyses, correct movement and correct search trials were taken into account. For the analysis of error rates in the search task incorrect movement trials were excluded, and for the analyses of error rates in the movement task, incorrect trials in the search task were excluded. Moreover, RT outliers (±3 SD from the overall mean RT of correct trials for each participant and each experimental block separately) were excluded from the RT analysis. Two separate ANOVAs with the factors congruency of movement (congruent vs. incongruent), display type (target present vs. absent), and block (1 vs. 2) were conducted for both mean RTs and error rates.

#### Analysis of pupil data

Pupil data were preprocessed to exclude blinks and other noise by means of a program developed by Henk van Steenbergen (Leiden University). It replaced missing values by the value measured for the other eye or, when data points were missing for both eyes, an interpolation between the pupil size of the last valid value before and the first valid value after the blink or otherwise missing data point.

For calculating the mean tonic pupil sizes for both experimental blocks, the mean values of data points recorded during a 600-ms interval directly preceding the movement cue onset of each trial (for similar procedure, see, e.g., Heitz et al., 2008) were calculated across all trials of block 1 and 2 separately. We chose an interval of 600 ms since it was identical to the minimum time the fixation mark stayed on the screen before each trial and because the baseline interval is commonly chosen in the range between 100 ms (e.g., Verney et al., 2004) and 1000 ms (e.g., Porter et al., 2007).

To assess the individual changes in motivation during the experimental session, we calculated the change in tonic pupil size (Δ_psize_) from Block 1 to Block 2 by subtracting, for each participant, the average trial-baseline pupil size in the latter from the average trial-baseline pupil size in the former. Positive values therefore, denote *decrease* in pupil size. Pearson correlations between Δ_psize_ and four other difference measures were analyzed: the change in overall performance across blocks [Δ_performance_ = overall mean RT (or error rate respectively) in block 2 minus overall mean RT (or error rate respectively) in block 1] and the change in congruency effect [Δ_congruency_ = congruency effect in Block 1 minus congruency effect in Block 2]. Congruency effects were calculated as follows: Mean RT (or error rate respectively) in congruent trials were subtracted from Mean RT (or error rate respectively) in incongruent trials. Also in these subtracted scores, positive values denote *decrease* in congruency effect. As it is a common pattern in visual search literature to find different effects for target present and target absent trials (see Chun and Wolfe, 1996 as well as Schubö et al., 2004, 2007), and as our previous results showed differential congruency effects for target present and target absent trials (Wykowska and Schubö, 2012; Wykowska et al., 2012, Experiment 2; Wykowska et al., 2009, Experiment 3), we considered only target present trials for correlational analyses.

### Results

#### Behavioral results

**Figure 2:**
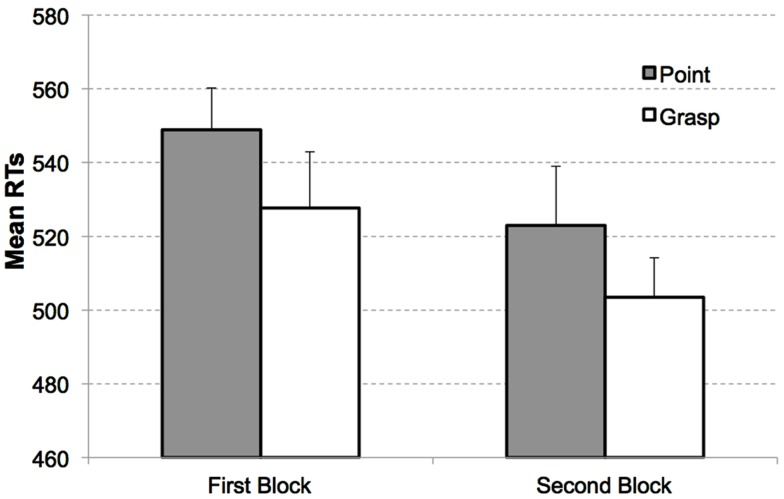
**Mean RTs as a function of congruency and block in Experiment 1**. Congruent (white bars) and incongruent condition (gray bars) for target present displays in the first block (left) and second block (right). Error bars indicate the standard errors of the mean, adapted to within-participants designs, according to procedure described in Cousineau (2005).

RTs from the search task were analyzed as a function of congruency, display type (target present or absent), and block (see Figure 2). A repeated measures ANOVA revealed significant main effects of congruency, *F*(1, 14) = 4.81, *p* < 0.05, $\eta_{P}^{2}=0.26$ with responses in the search task being faster for the congruent (*M* = 535 ms, SEM = 29 ms) than the incongruent condition (*M* = 549 ms, SEM = 34 ms); display type, *F*(1, 14) = 6.36, *p* < 0.05, $\eta_{P}^{2}=0.31$ with faster responses to target present displays (*M* = 526 ms, SEM = 34 ms) compared to target absent displays (*M* = 559 ms, SEM = 29 ms); and Block, *F*(1, 14) = 6.29, *p* < 0.05, $\eta_{P}^{2}=0.31$ with slower responses in the first (*M* = 563 ms, SEM = 29 ms) than the second block (*M* = 522 ms, SEM = 35 ms). None of the interactions reached the level of significance, all *F*s < 1, *p*s > 0.4.

The repeated measures ANOVA on error rates revealed only a significant main effect of display type, *F*(1, 14) = 11.72, *p* < 0.005, $\eta_{P}^{2}=0.46$ with a higher error rate in target present displays (Misses; *M* = 8.9%, SEM = 1.9%) than in target absent displays (False alarms; *M* = 2.8%, SEM = 0.8%). No other effects or interactions reached the level of significance, all *F*s < 2, *p*s > 1. The pattern of error rates however, was in line with the results in RT data: congruent trials yielded smaller error rates (*M* = 5.7%, SEM = 1.3) than incongruent trials (*M* = 5.9%, SEM = 1.1.); and therefore, there was no speed-accuracy trade-off observed.

#### Error rates in the movement task

Comparison of accuracy across the two types of movements revealed no difference in performance for pointing and grasping movements, *t*(14) < 1, *p* > 0.36 with pointing movements yielding 2.3% of errors on average and grasping movements 2.8% of errors on average.

#### Pupil data

Individual congruency effects and pupil sizes for each block separately are presented in Table 1.

**Table 1:** **Individual average pupil sizes and congruency effects in Block 1 and Block 2 of Experiment 1, sorted according to increasing pupil size**.

Block 1			Block 2		
Pupil size (mm)	RT congruency (ms)	Participant	Pupil size (mm)	RT congruency (ms)	Participant
2.57	−7.82	4	2.62	37.17	4
2.74	47.10	6	2.69	38.62	6
3.07	−17.77	3	3.08	−6.70	3
3.14	49.18	8	3.15	−0.05	10
3.18	12.85	10	3.20	−14.01	5
3.28	6.32	12	3.22	40.93	8
3.42	3.38	5	3.30	4.97	12
3.43	20.04	11	3.33	6.39	11
3.51	21.56	1	3.42	−16.09	1
3.56	0.80	13	3.53	−1.87	13
3.57	11.20	7	3.58	19.09	2
3.61	18.63	2	3.73	35.60	7
4.19	53.03	9	3.95	−5.97	9

*Congruency effects were calculated as Mean RT_incongruent_ − Mean RT_congruent_ and therefore positive values indicate expected congruency effects, while negative values represent inverse congruency effects*.

**Figure 3:**
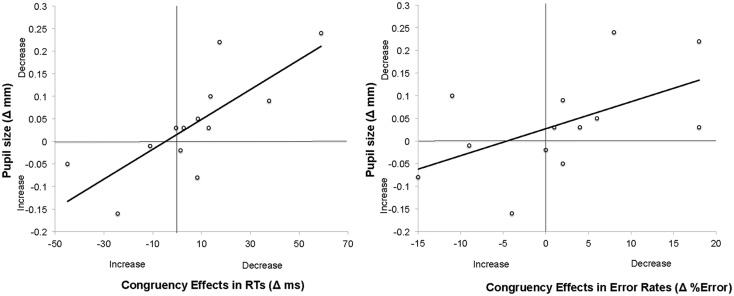
**Scatter plots and linear regression curves indicating the correlation between changes in pupil size and congruency effects (in RTs left, and in error rates right) across the two experimental blocks in Experiment 1**. The changes in pupil size were calculated as a Mean Pupil Size_Block1_ − Mean Pupil Size_Block2_. Therefore, positive values indicate *decrease* in pupil sizes. Changes in congruency effects were calculated as Congruency effect in RT/Error rate_Block1_ − Congruency Effect in RT/Error rate_Block2_. Positive values denote *decrease* in congruency effects across blocks.

Overall performance did not correlate with overall pupil size, *r*(13) = 0.001; *p* > 0.9. Similarly, changes in general level of performance (Δ_performance_) did not correlate with changes in tonic pupil size (Δ_psize_), neither for RT data, *r*(13) = −0.062; *p* > 0.8 nor for error rates, *r*(13) = 0.306; *p* > 0.3. Most importantly, however, Δ_psize_ was strongly correlated with changes in the congruency effect (Δ_congruency_) in both RTs, *r*(13) = 0.77, *p* < 0.005, and error rates, *r*(13) = 0.59, *p* < 0.05 (see Figure 3).

#### Median split analysis

In order to examine whether the effects of interest depended on individual differences in overall pupil size, we split the sample into two groups based on median pupil size (averaged across both experimental blocks). Pupil size (large vs. small) was then entered into a 2 × 2 mixed ANOVA as a between participants factor, with the within-participants factor of congruency. The analysis was conducted for target present trials only. Main effect of congruency was observed, *F*(1, 11) = 5.19, *p* < 0.05, but no interaction with pupil size, *F* < 0.2, *p* > 0.7. Subsequently, we tested whether the change in the congruency effect across blocks (Congruency_Block1_ − Congruency_Block2_) correlated with the change in pupil size (Pupil size_Block1_ − Pupil size_Block2_) for one of the groups more than for the other. Indeed, the correlation was significant only for the group of participants that had an overall larger pupil size, *r*(7) = 0.93, *p* < 0.01. The correlation was not observed for the group of participants with smaller pupil size *r*(6) < 0.55, *p* > 0.23. The change in overall performance (Mean RT_Block1_ − Mean RT_Block2_) did not correlate with the change in pupil size in either of the two groups, both *r*s < 0.45, *p*s > 0.4.

### Discussion

The aim of Experiment 1 was to examine the influence of individual differences in capacity for maintenance of motivation and effort throughout the experimental session on the size of action-perception congruency effects. We reasoned that losses of motivation/effort might affect the extent to which the required action plan is activated and this in turn might affect the intentional weighting mechanism, i.e., the action-related biases on perceptual selection of action-relevant characteristics. Results indeed showed that changes in pupil size – a marker of individual motivation/effort – correlated with changes in congruency effects. In particular, the more the pupil size decreased over time (reflecting a decrease in motivation/effort), the more the size of congruency effects decreased as well. This suggests that maintenance of motivation/effort throughout an experimental session has a specific impact on the degree to which action plans are activated and the mechanism of intentional weighting is employed.

However, pupil size is only an indirect measure of motivation/effort. Therefore, Experiment 2 was conducted with the aim of examining the relationship between individual capacity for motivation maintenance and congruency effects with a more direct measure of motivation.

## Experiment 2

Experiment 2 was conducted in order to test whether the correlation between fluctuations in levels of motivation/engagement throughout the experiment and the changes in size of the congruency effects would also be observed when a more direct measure of motivation is applied: the level of motivation as measured with a visual analog scale (VAS, see Bond and Lader, 1974; see also Kleih et al., 2010, for a similar methodology to assess individual levels of motivation).

### Method

The paradigm of Experiment 2 remained similar to Experiment 1 except that instead of measuring pupil size during the experiments, the VAS was administered before the Experiment, between Block 1 and Block 2 and at the end of Experiment.

#### Participants

Sixteen volunteers took part in the experiment (10 women, all right-handed, all with normal or corrected-to-normal vision, mean age: 23.9, range 20–29). Participants were not informed about the exact purposes of the experiment, received monetary compensation, and provided written consent regarding their participation.

#### Stimuli and apparatus

Stimuli were presented on a standard 17′′ CRT screen (100 Hz refresh rate) placed at a distance of 75 cm from an observer. Stimulus presentation was controlled by E-Prime presentation software (Psychology Software Tools, Pittsburgh, PA, USA). Participants were seated in central position relative to the midpoint of the screen.

Stimuli were similar to that of Experiment 1, except that the sizes were slightly different due to a different distance from the computer screen (Experiment 2 was conducted in a different lab than Experiment 1). The fixation mark extended 0.22° of visual angle, movement cues (see Figure 1), covered 12.24° × 17.13° of visual angle, and the circles of the visual search display were 1.43° each with the target extending 2.17° of visual angle. The visual search displays were presented on a light gray background, on three imaginary circular arrays with diameters of 4.54°, 11.18°, and 17.44° of visual angle. The movement-relevant cup was indicated by a yellow asterisk (0.44° of visual angle; CIE L*a*b color coordinates: 87/5/82), which could appear at one of three different positions on the screen (4.54° of visual angle above the lower border of the screen and 4.45° from the left/right border, or in the middle in horizontal axis). The cups that the movement was supposed to be executed on were identical to those of Experiment 1 and were positioned 20 cm below the three possible positions of the yellow asterisks.

Similarly to Experiment 1, participants were to indicate whether or not they had detected a target by pressing a mouse key with the index and middle finger of their dominant hand. The mapping between mouse keys and target/blank trials was balanced between participants. The movement task (pointing vs. grasping of a cup) was carried out with the non-dominant hand. The experimenter monitored the performed movements with a camera and coded their correctness online by means of a mouse key.

#### Procedure

Similarly to Experiment 1, participants attended a practice session to become familiar with the movement task (180 trials). The experiment proper was conducted no earlier than 1 day and no later than 2 days after the practice session. The practice session started with 30 trials for each of the movement types separately, followed by 120 trials in which both movements were performed, in a randomized order. The experimental session proper started with participants receiving oral and written instructions. Subsequently, the first visual analog scale (VAS1) was administered. This was followed by one warm-up session in which only the movements task was required (18 trials) and one practice session in which both movement and search task were executed (36 trials). Subsequently, the actual experiment was conducted with two experimental blocks (288 trials each). Between the two experimental blocks, VAS2 was administered, and at the end of the whole experiment (after Block 2), participants filled in VAS3. Trial sequence and timing were identical as in Experiment 1, and are depicted in Figure 1.

#### Visual analog scales

Participants were asked to fill in three VASs at the beginning of Experiment (VAS1), after Block 1 (VAS2), and after Block2 (VAS3). The VASs were administered on paper, and comprised of four items: Alertness, Attentiveness, Interest, and Motivation. Each of the items consisted in a 96 mm long scale between the following states:

- Alertness: Alert (left) ------- Drowsy (right)
- Attentiveness: Attentive (left) ------- Dreamy (right)
- Interest: Interested (left) ------- Bored (right)
- Motivation: Motivated (left) ------- Unmotivated (right)

The two extreme states for each of the first three items were taken from Bond and Lader (1974) while for the last item they were taken from Kleih et al. (2010) in a slightly modified manner: in Kleih et al. (2010) the authors used the terms “*extremely* motivated/unmotivated.” Participants indicated their state by marking one position on the line that best represented their subjective assessment of their state.

### Analysis

#### Behavioral data in the search task

For the analysis of RT data only trials with correct movement and correct search responses were taken into account. Furthermore, RT outliers (±3 SD from the overall mean RT of correct trials for each participant and each experimental block separately) were excluded. For the analysis of accuracy in the search task, only correct movement trials were subject to the analysis. Two separate ANOVAs with the factors congruency of movement (congruent vs. incongruent), display type (target present vs. absent), and block (1 vs. 2) were conducted for both RTs and error rates in the search task. Accuracy in the movement task was analyzed with a pairwise *t*-test for error rates in the pointing vs. grasping movement for trials with errors in the search task excluded.

#### VAS analysis

Individual participants’ responses in each VAS were calculated as the percent of the distance from the leftmost extreme of the scale to the rightmost extreme for each of the items of the VAS (Alertness, Attentiveness, Interest, Motivation). Therefore, the smaller the value, the nearer it is to positive state (alert, attentive, interested, motivated); the larger the value, the more the state is assessed as negative (drowsy, dreamy, uninterested, unmotivated). Change of each of these states throughout the Experiment was calculated as the difference between the VAS2 and VAS3 by subtracting the scores in VAS2 from VAS3. Therefore, positive values represent *decrease* in alertness/attentiveness/interest/motivational state, while negative values represent increase in alertness/attentiveness/interest/motivational state. The initial motivation of each participant was assessed with VAS1, individual scores for each item of VAS 1 are presented in Table 2.

**Table 2:** **Individual scores for each item of the VAS administered before the experiment**.

Before experiment				
Participant	Alertness	Attentiveness	Interest	Motivation
1	46.00	29.00	16.00	23.00
2	51.04	38.54	56.25	26.04
3	35.42	36.46	45.83	3.13
4	25.00	31.25	4.17	4.17
5	20.83	42.71	20.83	23.96
6	15.63	52.08	19.79	13.54
7	26.04	25.00	14.58	12.50
8	20.83	22.92	31.25	21.88
9	20.83	13.54	7.29	9.38
10	79.17	83.33	76.04	73.96
11	18.75	22.92	19.79	17.71
12	20.83	23.96	14.58	17.71
13	61.46	73.96	76.04	48.96
14	68.75	72.92	47.92	67.71
15	19.79	14.58	1.04	10.42
16	5.21	3.13	0.00	0.00

*The scores on VAS were calculated as a percent of distance from leftmost extreme of the scale (positive state) to rightmost extreme of the scale (negative state). Therefore, the smaller the value, the nearer it is to positive state (alert, attentive, interested, motivated); the larger the value, the more the state is assessed as negative (drowsy, dreamy, uninterested, unmotivated)*.

### Results

#### RTs

**Figure 4:**
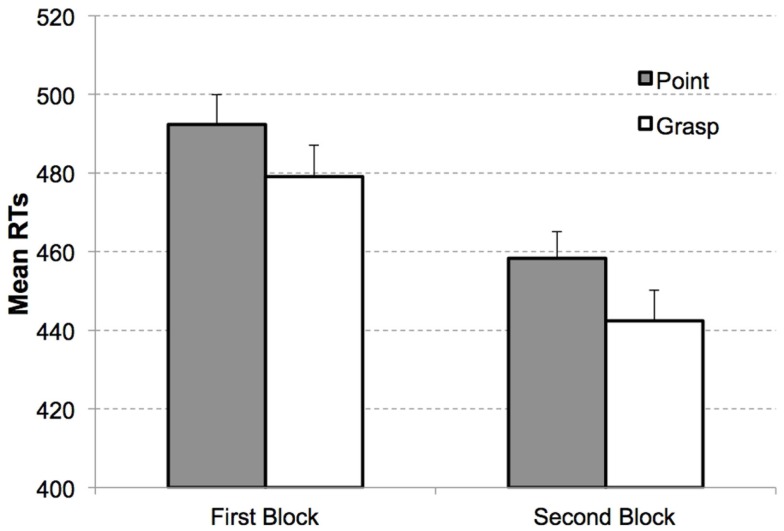
**Mean RTs as a function of congruency and block in Experiment 2**. Congruent (white bars) and incongruent condition (gray bars) for target present displays in the first block (left) and second block (right). Error bars indicate the standard errors of the mean, adapted to within-participants designs, according to procedure described in Cousineau (2005).

The 2 × 2 ANOVA on mean RTs with the factors movement type (grasping vs. pointing) and display type (target present vs. target absent) revealed a significant interaction between movement type and display type, *F*(1, 15) = 14.54, *p* < 0.005, $\eta_{P}^{2}=0.49$. No other effects reached the level of significance, all *p*s > 0.6. The congruency effect was observed for target present trials (*M*_Grasp_ = 461 ms, SEM = 17, *M*_Point_ = 475 ms, SEM = 16), *t*(15) = 1.94, *p* < 0.05, one-tailed, see Figure 4.

For target absent trials, the effect was reversed and also significant (*M*_Grasp_ = 459 ms, SEM = 18, *M*_Point_ = 477 ms, SEM = 18), *t*(15) = 4.25, *p* < 0.005, two-tailed. The differential congruency effects for target present vs. target absent trials are in line with previous findings (Wykowska and Schubö, 2012; Wykowska et al., 2012, Experiment 2; Wykowska et al., 2009, Experiment 3) and might reveal that when there is no target, the action-related weighting mechanism impedes (negative) responses, as the system needs to suppress activation of the pre-weighted dimension that is action-relevant. Moreover, it is a common pattern in visual search literature to find different effects for target present and target absent trials (see Chun and Wolfe, 1996, as well as Schubö et al., 2004, 2007).

#### Error rates in the search task

Analogous ANOVA on error rates also revealed a significant interaction between movement type and display type, *F*(1, 15) = 12.01, *p* < 0.005, $\eta_{P}^{2}=0.44$. There was also a significant main effect of display type, *F*(1, 15) = 10.52, *p* < 0.01, $\eta_{P}^{2}=0.41$ with larger error rates for target present trials (*M* = 8.1, SEM = 1.3) than for target absent trials (*M* = 2.9, SEM = 0.7); and a marginally significant effect of movement type, *F*(1, 15) = 3.43, *p* < 0.09, $\eta_{P}^{2}=0.18$, with slightly smaller error rates for the grasping movement condition (*M* = 4.9, SEM = 0.7) relative to the pointing condition (*M* = 6.1, SEM = 0.8). This effect was mainly driven by the target trials (*M*_Grasp_ = 6.4%, SEM = 1.2, *M*_Point_ = 9.82, SEM = 1.5), in which the congruency effect was significant, *t*(15) = 3.98, *p* < 005, two-tailed; and not by the target absent trials, which showed (similarly to RT data) a reverse pattern (*M*_Grasp_ = 3.5%, SEM = 1.05, *M*_Point_ = 2.3%, SEM = 0.49) that did not reach the level of significance, *t* < 1.5, *p* > 0.23, two-tailed. In sum, the pattern of results in search accuracy paralleled RT data, which speaks against any speed-accuracy trade-offs.

#### Error rates in the movement task

Comparison of accuracy across the two types of movements revealed no difference in performance for pointing and grasping movements, *t*(15) < 0.5, *p* > 0.6 with pointing movements yielding 2.2% of errors on average and grasping movements 1.9% of errors on average.

#### Visual analog scales

**Figure 5:**
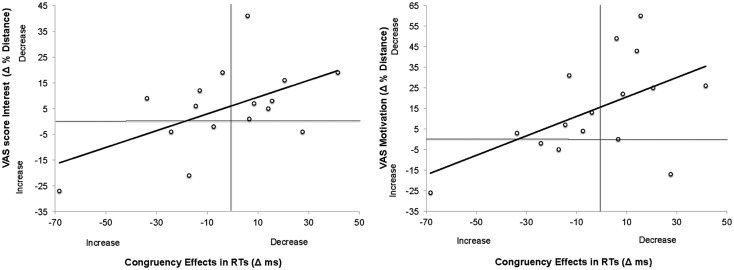
**Scatter plots and linear regression curves indicating the correlation between changes in VAS score on the item “Interest” and congruency effects (left); and between changes in VAS score on the item “Motivation” and congruency effects (right) across the two experimental blocks in Experiment 2**. The changes in VAS scores were calculated as Mean Score VAS3 − Mean Score VAS2. Therefore, positive values indicate *decrease* in pupil sizes. This is because the raw scores on VAS denoted the distance from the leftmost extreme (positive) to rightmost extreme (negative). Therefore, the smaller the numbers in raw scores, the more positive the state. Changes in congruency effects were calculated as Congruency Effect in RT/Error rate_Block1_ − Congruency Effect in RT/Error rate_Block2_. Positive values denote *decrease* in congruency effects across blocks.

Initial level of motivation in the task, as measured by any of the four items of the VAS, did not correlate with the size of the congruency effects, all *r*s < 0.15, *p*s > 0.6. However, similarly to the pattern of Experiment 1, the difference in VAS scores between two experimental blocks correlated with the difference in congruency effect in RT for two items on VAS, namely Item “Interest” *r*(15) = 0.542, *p* < 0.05 and Item “Motivation” *r*(15) = 0.528, *p* < 0.05 when the scores of VAS3 were subtracted from the scores of VAS2, see Figure 5.

The difference in two other items (Alertness, Attentiveness) did not correlate with difference in congruency effects across blocks, both *p*s > 0.32. Similarly, no significant correlations were observed for any of the items for differences between VAS1-VAS2, all *p*s > 0.24. The average of VAS scores for each of the items (averaged across scores on all three VASs) did not correlate with the overall average of congruency effects (averaged across both experimental blocks), all *p*s > 0.29. Individual congruency effects and scores for each VAS item and each block separately are presented in Table 3.

**Table 3:** **Individual congruency effects in Block 1 and Block 2 of Experiment 2, as well as individual scores on each of the items on the VAS scale administered after Block 2 and after Block 3; sorted according to increasing congruency effects**.

RT congruency (Δ ms)	Alertness	Attentiveness	Interest	Motivation	Participant
**BLOCK 1**					
−98.52	86.46	84.38	89.58	90.63	10
−26.06	54.17	20.83	14.58	14.58	7
−14.12	31.25	33.33	25.00	36.46	15
−13.19	10.42	14.58	15.63	16.67	11
7.19	15.63	35.42	35.42	19.79	6
14.26	8.33	7.29	0.00	1.04	16
15.49	31.25	38.54	58.33	4.17	3
16.20	11.46	21.88	33.33	6.25	4
19.48	38.54	43.75	38.54	38.54	12
22.54	22.92	18.75	17.71	18.75	2
29.05	26.04	11.46	18.75	11.46	9
31.46	47.92	39.58	39.58	23.96	1
37.49	0.00	16.67	26.04	11.46	5
37.53	76.04	58.33	35.42	21.88	14
47.99	44.79	36.46	42.71	36.46	8
84.91	28.13	17.71	43.75	28.13	13
**BLOCK 2**					
−41.69	15.63	16.67	20.83	19.79	15
−30.18	68.75	64.58	62.50	64.58	10
1.46	26.04	33.33	63.54	46.88	3
7.71	4.17	2.08	1.04	1.04	16
7.73	73.96	33.33	23.96	17.71	7
11.07	19.79	20.83	11.46	14.58	11
14.68	29.17	27.08	33.33	23.96	6
16.62	55.21	56.25	58.33	67.71	2
21.98	73.96	34.38	43.75	82.29	14
23.44	59.38	58.33	57.29	51.04	12
27.46	53.13	60.42	58.33	61.46	8
29.03	35.42	35.42	33.33	33.33	5
29.17	28.13	28.13	44.79	37.50	4
43.39	38.54	36.46	62.50	54.17	13
43.60	29.17	9.38	25.00	18.75	9
48.62	25.00	23.96	18.75	18.75	1

*Congruency effects were calculated as Mean RT_incongruent_ − Mean RT_congruent_ and therefore positive values indicate expected congruency effects, while negative values represent inverse congruency effects. The scores on VAS were calculated as a percent of distance from leftmost extreme of the scale (positive state) to rightmost extreme of the scale (negative state). Therefore, the smaller the value, the nearer it is to positive state (alert, attentive, bored, motivated); the larger the value, the nearer the state is assessed as negative (drowsy, dreamy, bored, unmotivated)*.

For differences in error rates, only correlation between the change in the item of “Motivation” and change in congruency effects in error rates approached the level of significance, *r*(15) = 0.456, *p* < 0.08 for VAS2–VAS3. All other correlations were not significant, all *p*s > 0.14.

#### Median split analyses

Similarly to Experiment 1, the data were analyzed as a function of small/large degree of overall motivation. To this end, the sample was split into two groups based on median score in the “Motivation” item of the VAS (averaged across VAS 2–3). Score on the VAS in the item “Motivation” (high vs. low) was entered into a 2 × 2 ANOVA as a between participants factor with congruency (congruent vs. incongruent) as a within-participants factor. The congruency effect did not interact with the “Motivation” score on VAS, *F* < 0.2, *p*s > 0.6. Also when the change in motivation (VAS2 − VAS3) was correlated with the change in congruency effect (Congruency_Block1_ − Congruency_Block2_) for each “Motivation” group separately (based on the median split), the analyses revealed that this correlation was not significant for either of the groups, *r*s < 0.5, *p*s > 0.2. The overall performance change (Mean RT_Block1_ − Mean RT_Block2_) did not correlate with change in VAS score on the item Motivation for either of the Motivation groups, *r*s < 0.4, *p*s > 0.3.

## General Discussion

The aim of the present study was to provide converging evidence, if possible, for a role of action control in visual attention and to further characterize that role by considering individual changes in motivation/mental effort over time. In line with the study of Wykowska et al. (2009, 2011, 2012), our participants showed better visual search performance if they had prepared a target-congruent manual action, that is, an action that relies on information from the target’s perceptual dimension. This effect was most likely related to the intentional weighting mechanism, which weighs higher processing of perceptual dimensions that are potentially relevant to the planned action. The interpretation that the intentional weighting mechanism biases perceptual processing with respect to action plans is in line with the idea that action and perception are tightly coupled (supported by results of, e.g., Müsseler and Hommel, 1997; Hommel, 1998; Craighero et al., 1999; Bekkering and Neggers, 2002; Fagioli et al., 2007; Wykowska et al., 2009, 2011, 2012). In more detail, intentional weighting allows for efficient delivery of perceptual information (such as shape/location of an object that is to be manipulated) to open parameters of online action control. This enables the action planning system to store only invariant parameters of particular actions in an offline representation, and to outsource specification of particular varying parameters to an efficient mechanism for selecting perceptual characteristics important for online control (Hommel, 2010; Wykowska et al., 2012; Memelink and Hommel, in press). Note that it is unlikely that the action-perception congruency effects could be explained by the assumption that one of the movements (grasping) was less demanding than the other movement (pointing), as (i) there were no differences observed in movement performance in either Experiment 1 or 2 (cf. analyses on movement error rates); (ii) in Experiment 2 there was an interaction between movement type and display type (target present vs. target absent trials), which speaks against the idea that grasping might have been less demanding and thereby facilitating search RTs in a non-specific manner; (iii) such interaction was already observed previously (Wykowska and Schubö, 2012; Wykowska et al., 2012, Experiment 2; Wykowska et al., 2009, Experiment 3); (iv) in previous studies (Wykowska et al., 2009; Wykowska and Schubö, 2012) there were two target dimensions introduced and the congruency effects consisted in an interaction between target dimension and movement type. For size targets, the grasping condition elicited better performance, while for luminance targets the pattern was reversed. If the observed effects were to be due to one of the movements being overall less demanding than the other, better performance in the search task would be observed in that movement condition for any target dimension, and for both target present and target absent trials, which was not the case.

Interesting for present purposes was the finding that this congruency effect varies with motivation/effort: decreases in pupil size (our motivational marker in Experiment 1) and in scores on VAS (probing subjective assessment of motivation and interest in the task in Experiment 2) across the two experimental blocks were accompanied by decreases in the size of the congruency effect in both reaction times (Experiment 1 and 2) and error rates (Experiment 1). As neither pupil size nor the overall score on VAS correlated with general search performance, this covariation cannot be attributed to the amount of general effort put into the perceptual task and/or attentional processes needed for the visual search task. Rather, maintenance of motivation/effort throughout the experiment presumably affected activation of the given movement representation, which in turn, might have determined the strength of the resulting attentional bias toward action congruent stimuli. In other words, the more effort people put and maintain in action preparation, the stronger the effects of action planning on perception. Although such interpretation needs further testing, this explanation is most plausible given the specific impact of changes in motivation on changes in congruency effects; and not on changes in overall performance. Furthermore, even though correlations need to be interpreted with caution, we take this as evidence for the idea that action planning affects perception and that motivation/effort modulates the strength of this impact. It might as well be the case that motivation has a general influence on attentional control settings (e.g., Folk et al., 1992) and not specifically on the action-related bias of perception. This explanation, however, does not exclude the idea that motivation had also an impact on motor preparation, which is closely linked with attention control settings (e.g., Hommel, 2010; Memelink and Hommel, in press).

It is interesting to note that *changes* in motivation/interest (as measured by changes in pupil size/scores on VAS) were better predictors of individual differences in the congruency effect than the overall levels motivation. There are several, not necessarily mutually exclusive reasons for why that might be the case. For one, it might be that individuals do not differ so much in the peak level of their motivation, which arguably was reached at the beginning of the experiment, but in the degree to which they can maintain that level for a longer time. Indeed, the range of scores on VAS 1 was 74% while on VAS 2 and VAS 3 it was 90% and 81% respectively. This is in line with previous findings showing that tasks that require the maintenance of a high level of readiness are particularly diagnostic for individual differences in task performance (e.g., Duncan et al., 1996). For another, motivation-independent inter-individual differences in pupil size are commonly very large (see, e.g., Winn et al., 1994), which could have obscured a more general relationship between motivational state and the congruency effect. This explanation, however, has not been confirmed by the analyses on two sub-samples sorted according to their overall pupil size based on a median split. In this analysis, the between-subjects factor of overall pupil size did not interact with congruency effects, indicating that the overall inter-individual differences in motivational state do not influence the congruency effects in general. Furthermore, note that even though inter-individual differences in pupil size affected the observed correlation between change in pupil size and change in congruency effects, this pattern was not observed in Experiment 2, when the sample was split based on the median score on the “Motivation” item of VAS. Hence, most probably, it is not that participants who have an overall higher degree of motivation are more likely to have congruency effects affected by the gradual loss of motivation. A more likely interpretation is that for those participants who have overall (motivation-independent) larger pupil size, this measure is more sensitive to capturing the relationship between loss of motivation throughout the experiment and size of the congruency effects, which might be in line with the law of initial values (Lacey, 1956) which assumes that the size of a physiological response to a stimulus might be affected by the baseline size of that response. In the case of the present study, the overall smaller pupil sizes were not decreasing further to the extent that larger pupil sizes were – a kind of a floor effect.

An alternative interpretation of tonic pupil size postulates that – in contrast to *phasic* pupil size that reflects selective processing/engagement in task, and an “exploitation” mode of control – *tonic* pupil size reflects general alertness and an “exploration” mode of control (Gilzenrat et al., 2010). Gilzenrat and colleagues claim that these two modes parallel the functionality of the locus coeruleus-norepinephrine (LC-NE) system (Rajkowski et al., 1993), which is highly correlated with pupil diameter. Typically, the *tonic* mode is associated with larger pupil size, higher general alertness but lower performance in a specific task – which permits exploratory behavior at the cost of lesser task engagement. In contrast, the phasic mode is correlated with smaller pupil size and higher performance allowing for better focus on a given task, at the cost of exploring the environment. In the present study we measured tonic pupil size, and decrease in pupil size was correlated with decreases in congruency effects. According to the LC-NE interpretation of tonic pupil size, this would mean that the decrease in alertness and *increase* in engagement in the task (as indicated in decrease in pupil size) would lead to decrease in congruency effects. Although at the first sight, this seems counterintuitive, Gilzenrat and colleagues point to an important aspect of the tonic mode of the LC-NE system, which would offer a plausible explanation of the present data in this theoretical context. The authors refer to the work of Hanoch and Vitouch (2004) as well as Zajonc (1980) and argue that because the exploratory mode is related to high alertness (and low focus on a specific task), the adaptiveness of such a mode consists most likely in readiness for efficient and quick action – at the cost of thorough or precise information processing. This would be in line with the present pattern of results. As congruency effects reflect a mechanism that biases perceptual processing for online action control (online adjustment of parameters), the system might be more likely to employ this mechanism in the high alertness-exploration mode than in the focused-exploitation mode. Therefore, the apparently counterintuitive interpretation of the changes of tonic pupil size in the context of the present data is well in line when one considers adaptiveness of the high alertness exploratory mode of the LC-NE system. This interpretation, however, has only a speculative character at present, and should be further tested.

The demonstrated interaction between motivation and congruency effects is not only of theoretical interest but could also be relevant for experimental practice. Even though it is commonly assumed that more extended testing provides a more reliable estimate of the cognitive processes under investigation, our observations suggest that relatively quickly occurring losses in motivation/effort can wash out and effectively eliminate actually existing effects as a function of time on task. Along the same lines, participants with greater losses of motivation/arousal/interest in the task might be expected to show weaker evidence of the effect under investigation which, among other things, questions the general practice of enforcing student participation in experiments through course credits. In any case, controlling for motivation/engagement might be generally advisable for experiments with surprising outcomes and failures to replicate. Using pupillometry as a tool to examine the fluctuations in the level of motivation/arousal/effort in various experimental procedures might therefore prove useful.

## Conflict of Interest Statement

The authors declare that the research was conducted in the absence of any commercial or financial relationships that could be construed as a potential conflict of interest.

## Footnotes

^1^Note that some of the behavioral data from the present Experiment 1 have been published in Wykowska et al. (2011). Importantly, this refers only to the congruency effects. The analyses presented here and the pupillometry data have not been published elsewhere.
